# Interim estimates of the effectiveness of the influenza vaccine against A(H3N2) influenza in adults in South Korea, 2016–2017 season

**DOI:** 10.1371/journal.pone.0178010

**Published:** 2017-05-25

**Authors:** Ji Yun Noh, Sooyeon Lim, Joon Young Song, Won Suk Choi, Hye Won Jeong, Jung Yeon Heo, Jacob Lee, Yu Bin Seo, Jin-Soo Lee, Seong Heon Wie, Young Keun Kim, Kyung Hwa Park, Sook-In Jung, Shin Woo Kim, Sun Hee Lee, Han Sol Lee, Young Hoon Yoon, Hee Jin Cheong, Woo Joo Kim

**Affiliations:** 1 Division of Infectious Diseases, Department of Internal Medicine, Korea University College of Medicine, Seoul, South Korea; 2 Asia Pacific Influenza Institute, Korea University College of Medicine, Seoul, South Korea; 3 Division of Infectious Diseases, Department of Internal Medicine, College of Medicine, Chungbuk National University, Cheongju, South Korea; 4 Division of Infectious Diseases, Department of Internal Medicine, Kangnam Sacred Heart Hospital, Hallym University School of Medicine, Chuncheon, South Korea; 5 Division of Infectious Diseases, Department of Internal Medicine, Inha University College of Medicine, Incheon, South Korea; 6 Division of Infectious Diseases, Department of Internal Medicine, The Catholic University of Korea, School of Medicine, St. Vincent's Hospital, Suwon, South Korea; 7 Department of Infectious Diseases, Yonsei University Wonju College of Medicine, Wonju, South Korea; 8 Department of Internal Medicine, Chonnam National University Medical School, Gwangju, South Korea; 9 Department of Internal Medicine, Kyungpook National University School of Medicine, Daegu, South Korea; 10 Department of Internal Medicine, Pusan National University School of Medicine, Busan, South Korea; 11 Brain Korea 21 Plus for Biomedical Science, Korea University College of Medicine, Seoul, South Korea; 12 Department of Emergency Medicine, Korea University College of Medicine, Seoul, South Korea; University of Hong Kong, HONG KONG

## Abstract

In the 2016–2017 season, the A(H3N2) influenza epidemic presented an unusual early peak pattern compared with past seasons in South Korea. The interim vaccine effectiveness (VE) of influenza vaccination in preventing laboratory-confirmed influenza was estimated using test-negative design through the tertiary hospital-based influenza surveillance system in South Korea. From 1 September, 2016 to 7 January, 2017, adjusted VE of influenza vaccination in preventing laboratory-confirmed A(H3N2) was -52.1% (95% confidence interval [CI], -147.2 to 6.4); -70.0% (95% CI, -212.0 to 7.4) in 19–64 years and 4.3% (95% CI, -137.8 to 61.5) in the elderly. Circulating A(H3N2) viruses belonged to the three phylogenetic subclades of 3C.2a, differently to A/Hong Kong/4801/2014, the current vaccine strain. Amino acid substitutions in hemagglutinin of circulating viruses seem to contribute to low VE. In conclusion, interim VE analysis presented that the protection of laboratory-confirmed influenza by seasonal influenza vaccination did not show the statistical significance in South Korea in the 2016–2017 influenza season.

## Introduction

Influenza has been considered major medically attended acute febrile respiratory illness in humans. Antigenic variation is responsible for influenza being a continuous concern for public health. The 2016–2017 influenza season in South Korea has been characterized by an exceptionally early epidemic of A(H3N2) influenza in December, 2016. A primary clinic-based sentinel surveillance conducted by the Korea Centers for Disease Control and Prevention (KCDC) reported that all the detected influenza viruses up to the week 1 (1 to 7 January, 2017) during the 2016–2017 influenza season were identified as A(H3N2) [1].

Vaccination is the primary strategy used to reduce the disease burden associated with influenza. Persons at a higher risk for influenza-related morbidity are recommended to be vaccinated against seasonal influenza with priority by KCDC in South Korea: persons who have chronic pulmonary disease or chronic heart disease; residents of nursing homes or long-term care facilities who have chronic medical diseases; persons who have chronic hepatic disease, chronic kidney disease, neuromuscular disorder, malignancy, diabetes mellitus, immunocompromised patients, children and adolescents (aged 6 months to 18 years) who are receiving aspirin therapy; persons aged ≥65 years; healthcare workers; household contacts of persons with chronic medical conditions, pregnant women and the elderly; caregivers of children aged <6 months; pregnant women; persons aged 50–64 years; children aged 6 through 59 months. The government provides free seasonal influenza vaccine to the eligible elderly aged 65 and more. In the 2016–2017 influenza season, national immunization program for free seasonal influenza vaccination extended to children 6 months to <12 months of age. Influenza vaccination coverage rate in general population and high-risk group was 34.3% and 61.3%, respectively, in the survey in 2007 in South Korea [2]. From October 2015 to January 2016, influenza vaccination coverage rate by the national immunization program in the elderly aged ≥65 was estimated as 81.0% [3]. During the 2016–2017 influenza season, egg-based trivalent influenza vaccine (TIV), cell-culture TIV, egg-based quadrivalent influenza vaccine (QIV), cell-culture QIV, and MF59-adjuvanted TIV were distributed in South Korea [3].

The estimation of influenza vaccine effectiveness (VE) is important to evaluate the impact of influenza vaccination programs and to assess the risk, especially in people at high risk for severe influenza and influenza-related complications. Early VE estimates may be useful to guide public health resource allocation or to conduct additional preventive measures if the VE is low [4].

In this study, we aimed to estimate interim VE in preventing laboratory-confirmed influenza (LCI) in the teaching hospital-based surveillance on influenza-like illness (ILI) patients in the emergency room (ER) and hospitalized patients with influenza. In addition, we investigated the molecular characteristics of circulating A(H3N2) influenza viruses during the early period in the 2016–2017 influenza season in South Korea.

## Methods

### Surveillance and study population

The study was conducted through the Hospital-based Influenza Morbidity and Mortality (HIMM), the 10 tertiary hospital-based influenza surveillance scheme in South Korea. The HIMM surveillance has been operated since the 2011–2012 influenza season. A total of seven teaching hospitals participated in the surveillance in 2011, which was extended to 10 hospitals in the 2012–2013 influenza season [5]. Participating hospitals were selected on the basis of geographic location, population served, and capacity of emergency department: two in Seoul, each one in Suwon, Ansan, Wonju, Incheon, Cheongju, Gwangju, Daegu, and Busan city.

The subjects of the surveillance were patients who visited the ER with ILI or those who were hospitalized with influenza presenting as ILI. Among ILI patients who visited ER, enrollment rate to the study varied season to season from 13.5% to 40.9%. In the ER, enrolled patients wrote clinical information including symptoms, underlying diseases and influenza vaccination on the self-report form. When the patients indicated unknown influenza vaccination status on the self-report form, researchers verified the vaccination history by asking and checking the medical records if available. Two respiratory specimens were collected according to a standard protocol. Rapid influenza detection test (RIDT) was performed at the bedside in ER and another nasopharyngeal swab samples were transported to the central laboratory in Korea University Guro Hospital for real-time, reverse-transcription polymerase chain reaction (RT-PCR) test. In addition, medical records were collected retrospectively from the hospitalized influenza patients.

From 1 September, 2016 to 7 January, 2017, patients over 19 years old who visited the ER with ILI or those who were hospitalized due to influenza presenting as ILI were enrolled in this study. ILI was defined as an acute respiratory illness with fever (measured body temperature of ≥38°C or self-reported by the patient) and at least one of the following symptoms, cough, sore throat, or rhinorrhea/nasal congestion, with an onset within the previous 7 days.

### Statistical analyses

The VE was estimated using the test-negative design. Patients identified as influenza-positive by PCR were considered as cases of LCI. The patients were considered to be vaccinated for the influenza if they received the vaccine at least 14 days before the onset of symptoms. Influenza B patients, patients with unknown vaccination status, and patients with influenza-negative by PCR and influenza-positive by RIDT were excluded from the analysis. Co-detection of influenza A and B cases were included in the analysis to estimate VE in preventing influenza A. The VE was calculated as [100 x (1-odds ratio for influenza in vaccinated compared to unvaccinated persons)]. Logistic regression models were adjusted for sex, age group (19–64 years and ≥65 years), calendar weeks (two-weeks interval), and comorbidities. Comorbidity was defined as any of following conditions: diabetes mellitus, cardiovascular disease, cerebrovascular disease, neuromuscular disease, chronic pulmonary disease, asthma, chronic kidney disease, chronic liver disease, malignancy, organ transplantation, bone marrow transplantation, immunosuppressant agent use, autoimmune disease, human immunodeficiency virus infection, and pregnancy. Age was adjusted as a continuous variable for age group-stratified analyses on VE. Statistical analyses were performed using SPSS version 20.0 (Chicago, IL, US).

### Molecular analyses

Nasopharyngeal swabs from twenty-nine A(H3N2) patients collected from week 50 to week 52 (4 to 24 December), 2016, were inoculated into Madin Darby canine kidney cells to isolate influenza viruses. Full length of hemagglutinin (HA) gene of the isolates was sequenced [6]. A distance-based maximum likelihood phylogenetic tree was generated using MEGA software v.6 with 1,000 bootstrap replicates and root to A/Perth/16/2009 [7]. Correlation plots were constructed using R packages displaying the positive/negative correlation between each amino acid substitution, which was indicative of classification of the subdivisions and subgroups of influenza viruses [8].

### Ethics statement

This study was approved by the Institutional Review Board in Korea University Guro Hospital (approval number: KUGH11088), Korea University Ansan Hospital (AS11047), Chungbuk National University Hospital (2011-06-044), Hallym University Kangnam Sacred Heart Hospital (2011-06-50), Inha University Hospital (11–1534), The Catholic University St. Vincent's Hospital (VC11ONME0118), Wonju Severance Christian Hospital (CR311025), Chonnam National University Hospital (CNUH-2012-133), Kyungpook National University Hospital (2012-07-030), and Pusan National University Hospital (1208-010-009). Written informed consent was obtained from the participants or their legal representatives.

## Results

### Characteristics of the study population

**Fig 1:**
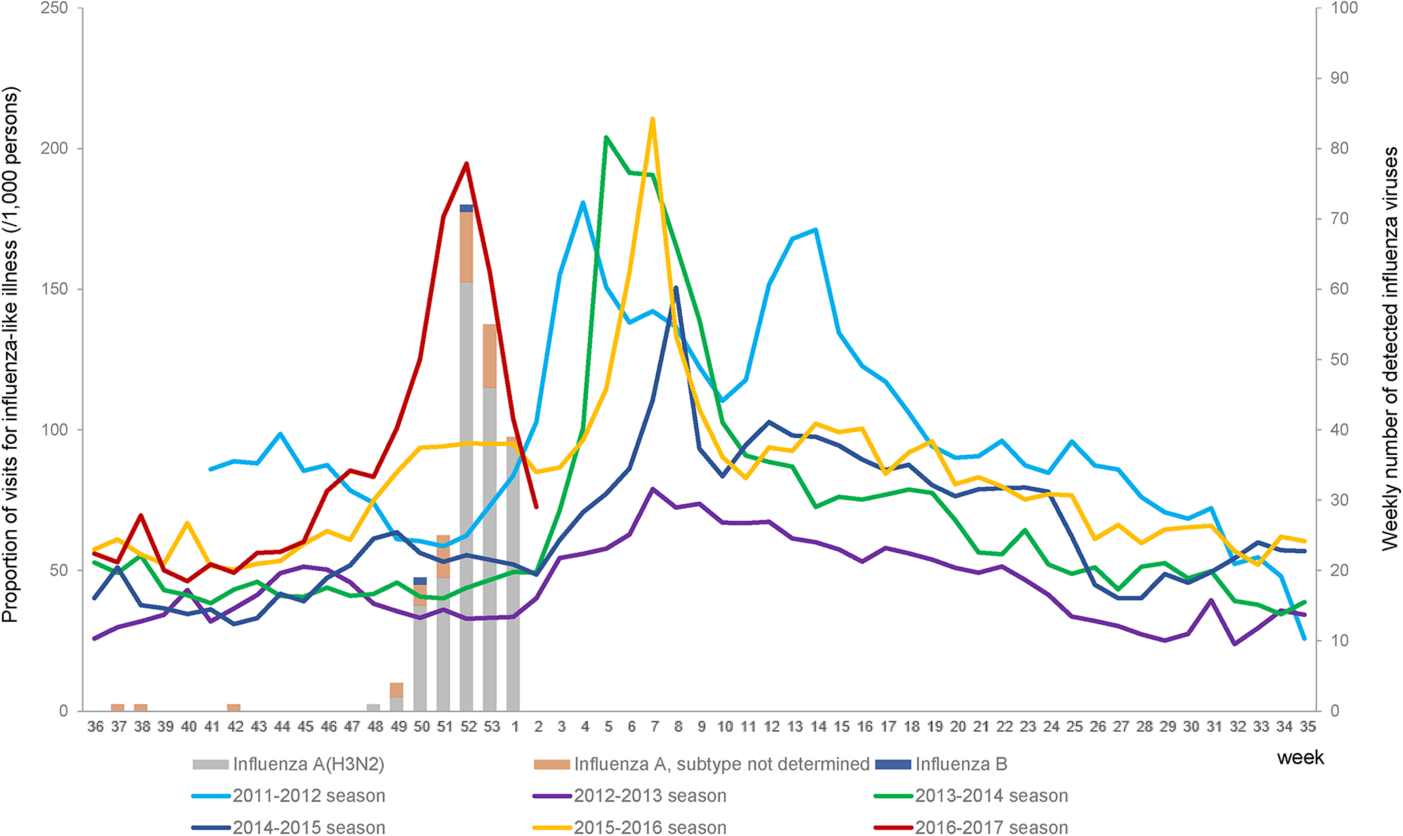
The proportion of visits for influenza-like illness per 1,000 persons in the emergency room of tertiary hospitals and the number of detected influenza viruses in 2016–2017 season through the hospital-based morbidity and mortality surveillance, South Korea.

Up to the week 1 of the 2016–2017 influenza season (1 to 7 January, 2017), the peak influenza activity was seen in week 52 (18 to 24 December, 2016) (Fig 1). During the study period, 492 patients with ILI were enrolled in the study. The 43 patients without PCR data and 1 patient of influenza B were excluded from the analysis. Patients with unknown vaccination status (n = 16), vaccination less than 14 days from onset (n = 11), and influenza-negative by PCR and influenza-positive by RIDT (n = 25) were excluded from the analysis. Finally, 400 ILI patients were eligible for the analysis.

The median age of the patients was 50 years (range, 19–94 years) and 126 (31.5%) patients were vaccinated against seasonal influenza during the 2016–2017 season. Among 400 patients, 52 (13.0%) were hospitalized. A total of 216 (54.0%) patients were diagnosed with influenza by PCR: A(H3N2), 180; influenza A, subtype not determined, 34; co-detection of A(H3N2) and influenza B, 2 cases. One or more comorbidities were found among 170 patients (42.5%). The demographic characteristics of the study population were shown on Table 1.

**Table 1:** Demographic characteristics of the study population (n = 400).

	Influenza positive (n = 216)	Influenza negative (n = 184)
Male, n (%)	87 (40.3)	75 (40.8)
Age		
19–64 years	158 (73.1)	119 (64.7)
≥65 years	58 (26.9)	65 (35.3)
Month of enrollment		
September, 2016	2 (0.9)	2 (1.1)
October, 2016	1 (0.5)	6 (3.3)
November, 2016	3 (1.4)	11 (6.0)
December, 2016	171 (79.2)	121 (65.8)
January, 2017	39 (18.1)	44 (23.9)
Comorbidity	83 (38.4)	87 (47.3)
Diabetes mellitus	32 (14.8)	31 (16.8)
Cardiovascular disease	18 (8.3)	25 (13.6)
Cerebrovascular disease	15 (6.9)	9 (4.9)
Neuromuscular disease	11 (5.1)	2 (1.1)
Chronic lung disease	5 (2.3)	12 (6.5)
Chronic obstructive pulmonary disease	11 (5.1)	11 (6.0)
Asthma	7 (3.2)	9 (4.9)
Chronic kidney disease	5 (2.3)	7 (3.8)
Chronic liver disease	6 (2.8)	5 (2.7)
Solid malignancy	19 (8.8)	16 (8.7)
Hematologic malignancy	-	6 (3.3)
Organ transplantation	1 (0.5)	-
Bone marrow transplantation	-	-
Autoimmune disease	1 (0.5)	1 (0.5)
Immunosuppressant agent use	8 (3.7)	5 (2.7)
Pregnancy	7 (3.2)	3 (1.6)
Human immunodeficiency virus infection	1 (0.5)	-
Influenza vaccination, 2016–2017 season	73 (33.8)	53 (28.8)
Influenza virus		
A/H3N2	180 (83.3)	-
Influenza A, subtype not determined	34 (15.7)	-
Co-detection of A/H3N2 and influenza B	2 (0.9)	-

### Influenza vaccine effectiveness

There was no difference in the influenza vaccination rate between influenza cases and controls. The adjusted VE of the influenza vaccine was estimated as -35.6% (95% confidence interval [CI], -114.7 to 14.3) for influenza A and -52.1% (95% CI, -147.2 to 6.4) for A(H3N2) influenza. The age group-specific adjusted VE in preventing A(H3N2) influenza was -70.0% (95% CI, -212.0 to 7.4) in 19–64 years age group and 4.3% (95% CI, -137.8 to 61.5) in ≥65 years age group (Table 2).

**Table 2:** Estimated effectiveness of the influenza vaccine for preventing laboratory-confirmed influenza in adults from 1 September, 2016 to 7 January, 2017 in South Korea.

	Influenza positive Vaccinated/total (%)	Influenza negative Vaccinated/total (%)	p^a^	Adjusted VE (95% CI) (%)
Influenza A^b^				
Overall	73/216 (33.8)	53/184 (28.8)	0.33	-35.6 (-114.7 to 14.3)
19–64 years	47/158 (29.7)	23/119 (19.3)	0.05	-67.1 (-202.9 to 7.9)
≥65 years	26/58 (44.8)	30/65 (46.2)	1.00	22.7 (-71.8 to 65.2)
A(H3N2)				
Overall	64/182 (35.2)	53/184 (28.8)	0.22	-52.1 (-147.2 to 6.4)
19–64 years	43/143 (30.1)	23/119 (19.3)	0.06	-70.0 (-212.0 to 7.4)
≥65 years	21/39 (53.8)	30/65 (46.2)	0.54	4.3 (-137.8 to 61.5)

^a^Fisher’s exact test was performed.

^b^There were 34 influenza A cases of which subtypes were not determined due to lack of specimens.

### Molecular characteristics of A(H3N2) influenza viruses

**Fig 2:**
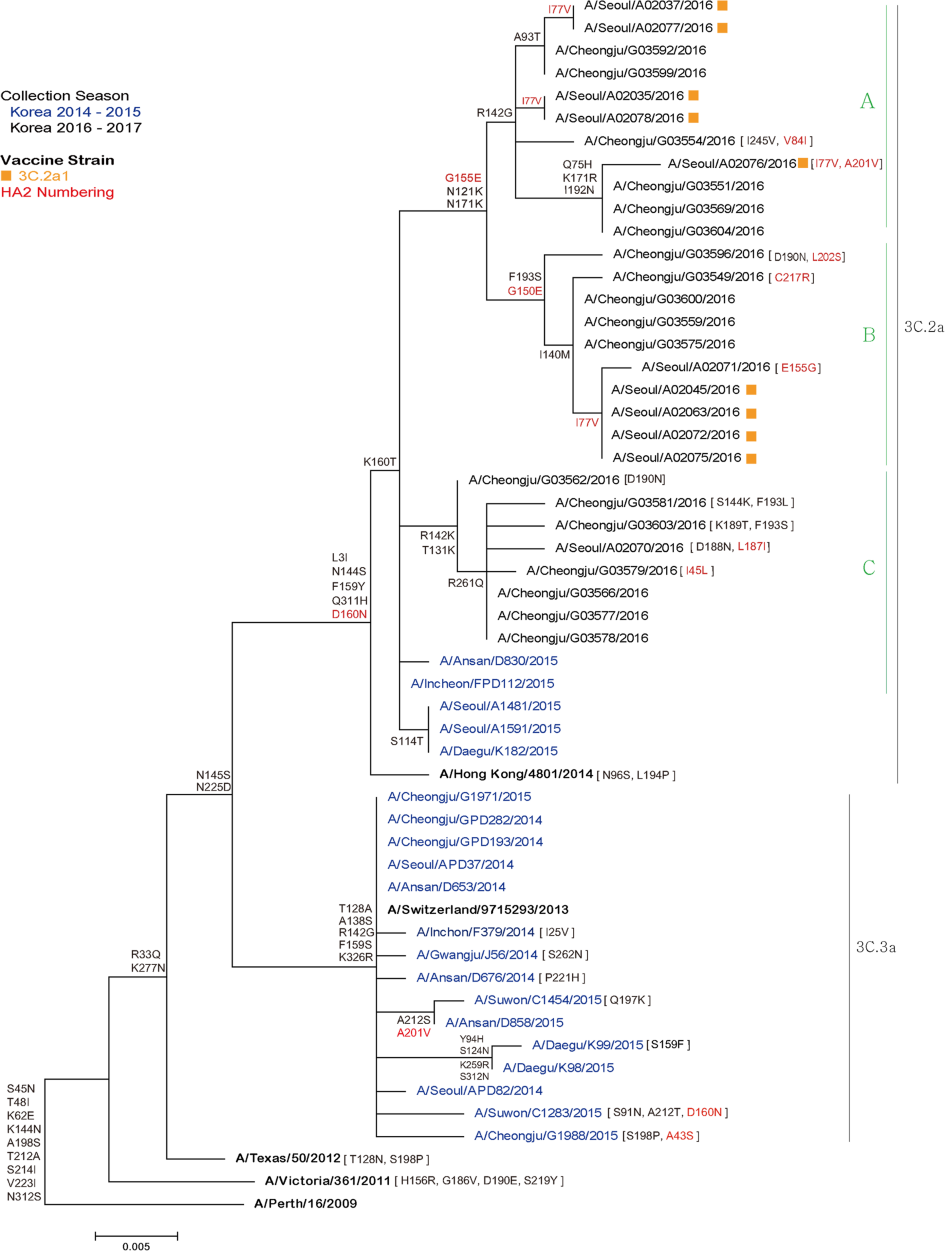
Phylogenetic analysis of A(H3N2) influenza viruses collected from week 50 to week 52, 2016 in South Korea. The phylogenetic tree was generated with the recent influenza vaccine composition strains for A(H3N2) in the Northern Hemisphere and isolates in the 2014–2015 season in South Korea. The reference sequences of vaccine strains were obtained from the EpiFlu database of the Global Initiative on Sharing Avian Influenza Data (GISAID) for A/Hong Kong/4801/2014 (EPI741474), Korea Influenza Sequence & Epitope Database (KISED) for A/Switzerland/9715293/2013, and GenBank for A/Texas/50/2012 (KC892248), A/Victoria/361/2011(KC306165) and A/Perth/16/2009 (KP457178).

Phylogenetic tree analysis revealed that the isolated A(H3N2) influenza viruses during the 2016–2017 influenza season belonged to the subclades of 3C.2a. However, all the 29 A(H3N2) influenza viruses fell to the another subclades, different from the A/Hong Kong/4801/2014 virus, the current vaccine strain, being designated by group A, B, and C (Fig 2). Viruses characterized with N121K, N171K, and G155E (HA2 numbering) were divided into two subclades depending on the R142G (group A), and F193S and G150E (HA2 numbering) (group B) mutations in there HA sequence. A(H3N2) influenza viruses of group C were characterized by R142K and T131K mutations in HA within the 3C.2a clade.

**Fig 3:**
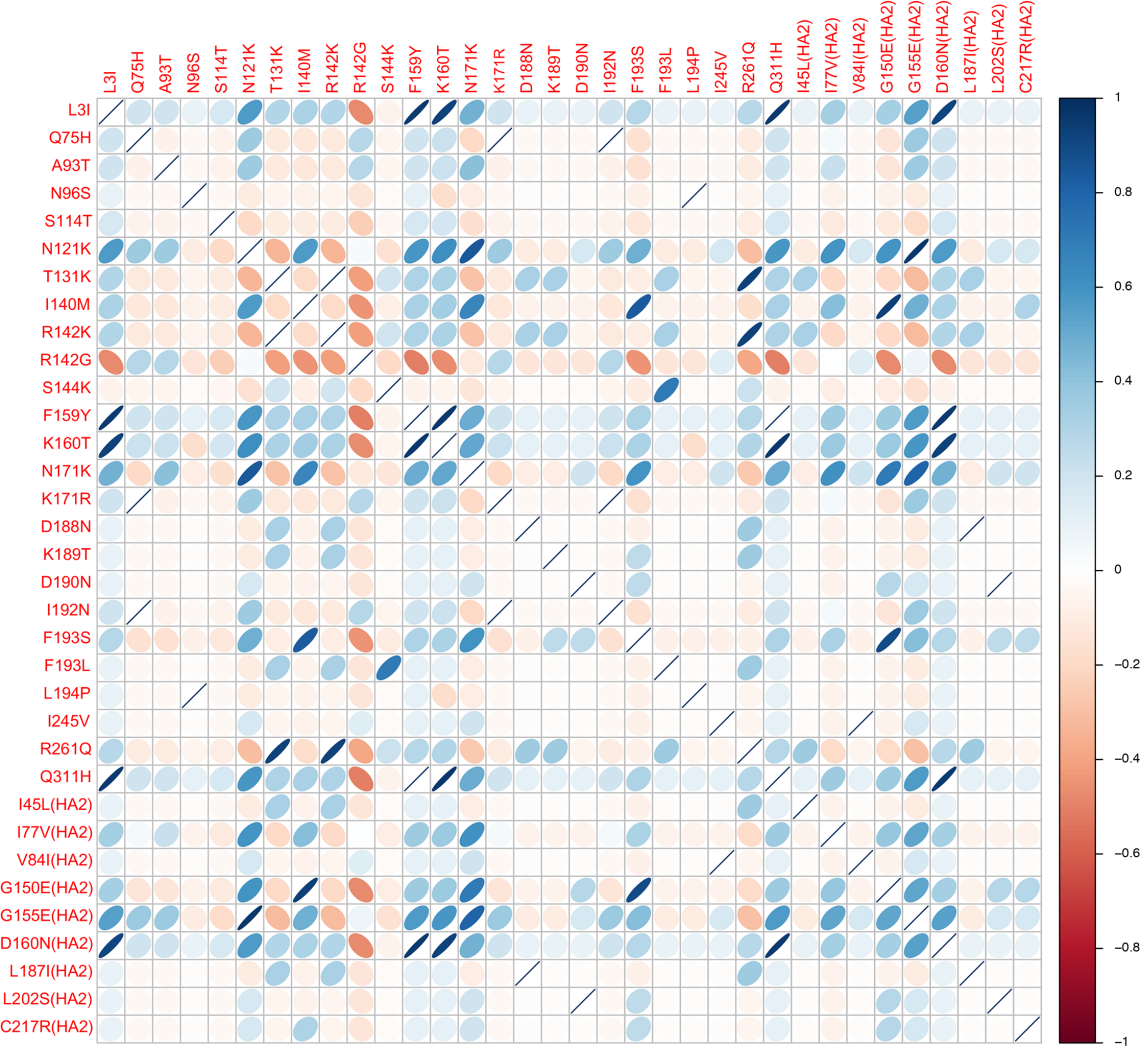
Correlation plot among 34 amino acid substitutions found in A(H3N2) influenza viruses within 3C.2a clade. Pearson’s correlation was calculated using R packages. Correlation coefficients were presented according to the value on the plot. The shapes and colors indicate the direction and strength of correlation. Blue color indicates a positive correlation, whereas red color represents negative correlation. The closer the shape of the ellipse shows linear mark, the closer the correlation value indicates to +1 or -1.

A correlation plot using 34 amino acid substitutions in HA from our isolates revealed that the R142K and R142G substitutions were positively and negatively correlated with the D160N substitution, respectively, suggesting that the R142K and R142G were obvious characteristics of viruses in the new subgroup (Fig 3).

## Discussion

In the 2016–2017 season, the A(H3N2) influenza epidemic showed an unusual early peak pattern compared with past seasons in South Korea. Also, the peak of influenza epidemic in week 52, 2016, was rather earlier than those of neighboring countries in Asia. Influenza laboratory surveillance information by the Global Influenza Surveillance and Response System showed the peak of number of specimens positive for influenza in week 3, 2017 in Japan and in week 1, 2017 in China [9].

In our study, the interim VE in preventing A(H3N2) influenza during the early 2016–2017 season was as low as -52.1% in adults. The circulating A(H3N2) viruses belonged to the 3C.2a subclades; however, some amino acid substitutions in the HA sequence of the clinical isolates caused them to build distinguishing subclades separately from the current vaccine strain in the phylogenetic tree.

Recent studies on the interim analysis of VE showed suboptimal influenza VE in preventing A(H3N2) influenza during the 2016–2017 season. In the US, influenza vaccine was effective to prevent A(H3N2) influenza presenting VE of 43% (95% CI, 29 to 54) in overall aged population, however, in 18–49 years and ≥65 years age group, protection did not reach statistical significance with wide range of confidence interval [10]. In northern Spain, VE for prevention of A(H3N2) influenza was 48% (95% CI, -1 to 65) in primary healthcare patients and 0% (95% CI, -38 to 27) in hospitalized patients [11]. In Canada, adjusted VE against influenza A(H3N2) was 42% (95% CI, 18 to 59) with variation by province and the distribution of clade 3C.2a1 A(H3N2) influenza variants was different by province [12]. In a multicenter case-control studies in Europe, early VE against A(H3N2) influenza was estimated as 2.0% (95% CI, -51.7 to 36.8) among ≥65 year-old at hospital level [13]. Meanwhile, in a register-base surveillance of VE against LCI in Sweden and Finland, mid-season analysis showed significant protection in persons aged ≥65 years [14]. About 60%-85% of circulating A/H3N2 influenza viruses belonged to the 3C.2a1 clade in those studies [10–12, 14].

Previous report referred A(H3N2) virus having changes of N171K, I406V, and G484E amino acid substitutions in the HA to as subclade 3C.2a1. Subclade 3C.2a1 viruses are defined with amino acid substitutions of 3C.2a plus N171K in HA1, I77V and G155E in HA2, and often N121K in HA1 as well [15]. Ferret antisera against A/Hong Kong/4801/2014 inhibited a majority of viruses in subclades 3C.2a1 [16]. However, in our study, viruses with N121K, N171K, and G155E (HA2 numbering) amino acid substitutions were divided into two subclades by R142G, and F193S and G150E (HA2 numbering) amino acid substitutions, irrespective of the existence of I77V mutations. In addition, viruses with R142K and T131K substitutions fell to another distinct subclade within 3C.2a presenting the strong positive correlation among R142K, T131K, and R261Q amino acid substitutions by the correlation plot. Among the 29 isolated A(H3N2) viruses, 11 (37.9%) had R142G substitution, which is one of the key characteristics of 3C.3a clade represented by the A/Switzerland/9715293/2013 virus, the vaccine strain in the 2015–2016 season. In the recent analysis of HA sequences of A(H3N2) influenza strains during the 2016–2017 season in London, co-circulation of multiple clades (3C.3a, 3C.2a, and 3C.2a1) was demonstrated and most variants belonged to a novel subclade which was proposed as 3C.2a2 [17]. Further research is required to investigate the impact of certain amino acid mutations of influenza viruses on the VE.

There are several limitations in this study. First, the sample size is small, as this study is an interim analysis. However, we tried to improve the reliability of our results by including two more weeks after the peak of the early influenza season into the study period. Second, surveillance was performed in tertiary hospitals and the study population is not representative of the population in South Korea. There is a chance that patients with more severe illness could gather to the tertiary hospitals. Therefore, it is possible that the VE estimates are different from those obtained in primary care clinic-based surveillance. However, there is an observation about non-heterogeneity between VE estimates in outpatient settings and inpatient settings [18]. Third, influenza vaccination data were obtained by self-report of patients and information of vaccination in the prior influenza season was not collected. Children and adolescent under 19 years old were not included in this study. In addition, virologic experiments to reveal the antigenic characteristics of circulating A(H3N2) viruses were not performed in this study. Further research is required to investigate the cross-reactivity between circulating viruses and the vaccine strain.

However, this study is noteworthy in estimating the low VE against A(H3N2) influenza in the early period of the 2016–2017 season and in characterizing the genetic features of circulating A(H3N2) viruses in South Korea. Recently, A(H3N2) mismatches between the vaccine strains and circulating strains have raised the global concern about suboptimal VE [19–21]. More active collection of epidemiologic and virologic data of influenza would contribute to better selection of vaccine strain. Furthermore, the selection of vaccine strains well matched with the expected epidemic strains and the development of broad spectrum influenza vaccines that cover intrasubtypic drift strains would both contribute to reducing the disease burden of influenza.
